# Self-Assembled Ru(II)-Coumarin Complexes for Selective Cell Membrane Imaging

**DOI:** 10.3390/pharmaceutics14112284

**Published:** 2022-10-25

**Authors:** Jiyin Liu, Xiaochun Xie, Junna Lu, Yi He, Dan Shao, Fangman Chen

**Affiliations:** 1 National Engineering Research Center for Tissue Restoration and Reconstruction, South China University of Technology, Guangzhou 510006, China; 2 School of Chemistry, Sun Yat-sen University, Guangzhou 510006, China; 3 School of Medicine, South China University of Technology, Guangzhou 510006, China; 4 Department of Rheumatology and Immunology, The Third Affiliated Hospital, Southern Medical University, Guangzhou 510665, China; 5 CAS Key Laboratory of Bio Medical Diagnostics, Suzhou Institute of Biomedical Engineering and Technology Chinese Academy of Sciences, Suzhou 215163, China

**Keywords:** cell membrane, imaging, self-assembly, nanoparticle, ruthenium

## Abstract

The cell membrane, as the protecting frontier of cells, is closely related to crucial biological behaviors including cell growth, death, and division. Lots of fluorescent probes have been fabricated to monitor cell membranes due to their simplicity and intuitiveness. However, the efficiency of those traditional probes has been limited by their susceptibility to photobleaching and poor water solubility. In this study, we have reported Ru(II)-coumarin complexes consisting of ruthenium, 1,10-phenanthroline, and coumarin 6 to further form self-assembled nanoprobes, for cell membrane targeting and imaging. The fluorescent property could be switchable from red to green through the dynamic disassembly of nanoprobes. Compared with commercial Dil, biocompatible nanoprobes exhibited superior stability for long-term cell imaging, along with remarkedly reduced background interference. Therefore, our self-assembled nanoprobe provides a powerful solution for investigating lipid trafficking with optical imaging.

## 1. Introduction

Cells are sophisticated systems, mainly consisting of a membrane, cytoplasm, and a nucleus. The membrane, the protecting frontier of cells, not only brings a physical interface between interiors and environment but also identifies the structure and function of organelles [1,2]. Additionally, many crucial biological behaviors including cell growth, death, and division rely on the membrane’s structure [3,4,5]. These biofunctions are accurately realized by several sophisticated components in the framework such as lipids, proteins, and carbohydrates [6,7,8,9]. There is strong desire for developing auxiliary tools to decipher the character of the membrane in physiological and pathological processes [10]. Recently, lots of fluorescent probes have been fabricated to monitor the cell membrane for their superior simplicity and intuitiveness [11,12]. For example, commercial dyes such as DiD, Dil, DiO, and DiR have been widely employed for cell membrane imaging with multicolor properties [13,14]. However, the efficiency of these traditional probes has been limited by their susceptibility to photobleaching and poor water solubility [15]. Despite the fact that some dyes with superior staining membrane performance have been developed recently, concerns regarding inefficient staining, poor photostability, and longer incubation times still need to be addressed [16,17].

Given the obvious limits of current imaging probes and the possible advantages of nanoparticles for diagnosis and detection, studies on nanotechnology for biomedical imaging represent a growing field [18,19]. Nanoparticles have promising benefits on improving biomedical imaging due to their unique targeting behavior, physicochemical properties, and superior stabilities [20,21]. Nevertheless, most nanoparticulate probes fail to light cell membranes due to their easier and fast internalization [22,23]. Therefore, there is a need for developing sophisticated nanomaterials to overcome these shortcomings for selective and stable cell-membrane imaging. Ruthenium (II) polypyridine complexes are one of the most extensively reported systems in the family of luminescent transition-metal complexes [24,25,26], making them interesting as fruitful probes for cellular imaging. Recently, several Ruthenium (II) complexes have been introduced for cell membrane-targeted imaging and therapy [26,27]. However, those luminescent probes lack efficient cell membrane retention and long-term imaging. Deep understanding of the structure-activity relationship regarding cell membrane selectivity will benefit the rational designed nanoprobes for efficient cell membrane imaging.

Herein, Ru(II)-coumarin complexes were designed and synthesized consisting of ruthenium, 1,10-phenanthroline and coumarin 6 to further form self-assembled nanoprobes for cell membrane targeting and imaging. The physicochemical and optical properties were characterized and compared between Ru(II)-coumarin complexes and nanoprobes. Then, the effect of Ru(II)-coumarin nanomaterials on the internalization and imaging of tumor cells and normal cells was investigated. The cytotoxicity was further evaluated by CCK-8 assay. The findings suggest that self-assembled Ru(II)-coumarin complex may be a highly effective nanoprobes for efficient cell membrane imaging.

## 2. Materials and Methods

### 2.1. Chemicals and Reagents

Coumarin 6 (purity: >94%), CCK-8, methyl sulfoxide, 1,10-phenanthroline, Ruthenium(III) chloride, lithium chloride and dimethylformamide were purchased from Sigma-Aldrich (St. Louis, MO, USA). Dulbecco’s Modified Eagle Medium (DMEM), fetal bovine serum (FBS), trypsin and penicillin-streptomycin (10,000 U/mL) were obtained from GIBCO (Carlsbad, CA, USA). The 4’,6-diamidino-2-phenylindole (DAPI) were purchased from Thermo Fisher Scientific (Waltham, MA, USA). All reagents were directly used without any further purification.

### 2.2. Preparation and Characterization of Ru(II)-Coumarin Complexes

To a mixture of RuCl_3_ (515.9 mg, 3 mmol) and 1,10-phenanthroline (1081.3 mg, 6 mmol) in 10 mL of dimethylformamide was added a catalytic amount of LiCl. The mixture was then refluxed at 100 °C for 24 h. After solvent removal, the residue was purified with column chromatography. The product was purplish red powder. Then the precursor and coumarin 6 was refluxed at 80 °C in ethanol for 24 h. The solvent was removed and the product was further purified with column chromatography, affording Ru(phen)_2_Cur as a dark brown powder. (850.5 mg, yield 35%). ^1^H NMR (400 MHz, d6-DMSO) δ 10.69 (d, 4H), 9.71(d, 4H), 8.72(d, 1H), 8.55(d, 1H), 8.17(t, 4H), 8.09 (s, 1H), 7.73 (d, 2H), 7.49(d, 4H), 6.96 (d, 1H), 6,18 (d, 1H), 6.03 (d, 1H), 2.02 (p, 4H), and 0.99 (t, 6H).

### 2.3. Preparation and Characterization of RuCM NP

To obtain nanoparticles of the Ru(phen)_2_Cur, a stock solution (40 mg/L in DMSO) was added to pure water (1:200, *v/v*) and then centrifuged to separate the nanoparticles. The UV-Vis absorption spectra of NPs (0.25 mM) were measured in water, while molecules (0.25 mM) were measured in DMSO with Shimadzu UV-1280. The fluorescence emission spectra of NPs and molecules were obtained using FLS 920 fluorescence spectrometer (Edinburgh Instrument, Livingston, UK) with 450 nm excitation. The quantum yield of the probe was measured by absolute quantum efficiency measurement system C9920-03G. The lifetime of the probe was measured on a FLS 980 fluorescence spectrometer (Edinburgh Instrument) with 471 nm excitation. All experiments were performed in quartz cuvettes (1 cm) at 25 °C.

### 2.4. Cell Culture and Selective Cell Membrane Imaging

Breast cancer cell line (4T1) was cultured in DMEM medium supplemented with 10% Fetal Bovine Serum (FBS) and 100 U/mL penicillin and streptomycin in a 5% CO_2_ incubator at 37 °C.

4T1 cells were installed in a 35 mm Petri dish and cultured 24 h. Then the Ru(II)-coumarin complexes (2.5 μg/mL) was added and co-incubated with cells for 4 h. Then, the cells were strained with Dil (a commercial cell membrane dye), and DAPI (nuclear dye) after rinsing three times with phosphate buffer solution to remove Ru(II)-coumarin complexes. Finally, the redundant dye was rinsing before imaging with inverted fluorescence microscope (Nikon Ti2-U, Nikon, Minato City, Japan) and the 361–389 excitation filter and 430–490 barrier filter were used for DAPI, the 464–495 excitation filter and 512–558 barrier filter were used for Ru(II)-coumarin complexes and the 540–580 excitation filter and 600–660 barrier filter were used for Dil. For photostability studies, the cells were stained with Ru(II)-Coumarin complexes (2.5 μg/mL) and Dil irradiated under 480 nm laser and 630 nm laser for 100 scans (1086 s).

### 2.5. Cytotoxicity

4T1 cells were inoculated into 96-well plates with a density of 5 × 10^3^ cells per well. Cells were cultured with 100 μL culture medium after 24 h. Different concentration of Ru(II)-Coumarin complexes, dissolved in a mixture of growth medium/DMSO (99.5:0.5, *v/v*), were added and culture with cells. After the 24 h, 100 μL culture medium were refreshed and 10 μL CCK-8 solution were add in per well. SpectraMax@ iD3 microplate reader was used to test the absorbance of 450 nm after incubating 1 h. Cell viability was calculated by the following equation:

Cell viability (%) = (mean OD value of treatment group/mean OD value of control group) × 100.

### 2.6. Statistical Analysis

All experiments were performed at least three times, and the results were exhibited as mean ± standard deviation. Independent student’s *t*-test (two groups) or Bonferroni’s post hoc test (three groups or more) were conducted to test for differences between groups. All analyses were carried out using SPSS software. *p*-values less than 0.05 were considered statistically significant.

## 3. Results and Discussions

**Figure 1:**
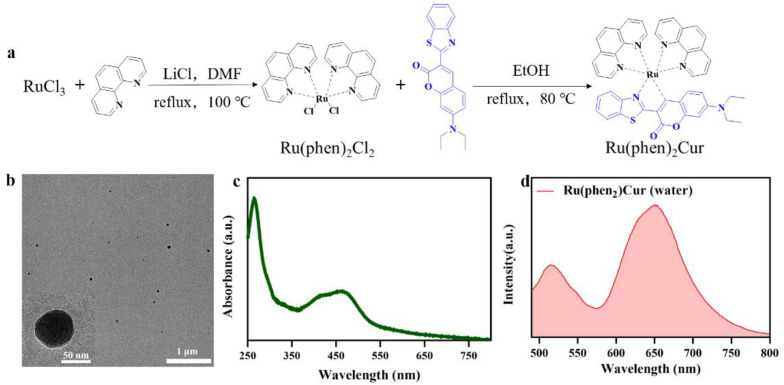
Scheme of Ru(II)-coumarin complexes preparation and characterization of RuCM NP. (**a**) Scheme of Ru(II)-coumarin complexes preparation. (**b**) TEM images of RuCM NP. Scale bars are 1 μm and 50 nm (inset), respectively. (**c**) UV-Vis absorption spectra of RuCM NP. (**d**) Fluorescence spectra of RuCM NP with the λ_ex_ = 450 nm.

The commercial dyes for cell membrane imaging probes usually bear two lipophilic carbon chains. The lipophilic chains facilitated the hydrophobic-hydrophobic interactions with phospholipid in the cell membrane. However, the strong hydrophobicity of the traditional dyes required cytotoxic organic solvents to dissolve the agents for storage. Therefore, the development of the water-soluble probes specifically targeting cell membrane was highly desired. Herein, we have designed and synthesized Ru(II)-coumarin complexes (Figure 1a), and its chemical structure was characterized by ^1^H NMR (Supplementary Figure S1).

We hypothesized that RuCM NP will interact with cell membranes due to their strong hydrophobicity and positive charge. The interaction will induce that RuCM NP nanoprobes disassembled to free molecules, which could insert into cell membranes for selective fluorescence imaging. To prove our hypothesis, the physicochemical properties of the probe were investigated in water and DMSO. The nanoprobes named RuCM NPs were prepared by rapidly adding DMSO solution containing Ru(II)-coumarin complexes into excessive volume of deionized water (Figure 1b). During the nanoprecipitation process, Ru(II)-coumarin molecules aggregated to form nanoparticles via intermolecular interactions, such as π−π stacking and hydrophobic interactions [28,29]. Based on the characterization via TEM (Figure 1b), the prepared RuCM NPs with a spherical structure (diameter: 56 ± 7.78 nm), which indicated the hydrophobicity of the free molecule. While dissolved in non-polar solvents, the Tyndall effect of the solution vanished immediately. (Supplementary Figure S2). Furthermore, disassembled nanoparticles could not be collected by high-speed centrifugation. Together, the phenomena indicated that probe dispersed in DMSO as free molecules. Zeta potential measurements further displayed that the RuCM NP exhibited a positive charge (37.38 ± 0.71 mV). The strong hydrophobicity and positive charge of RuCM NP confirmed the feasibility of the interaction with non-polar hydrocarbon chains of phospholipids.

**Figure 2:**
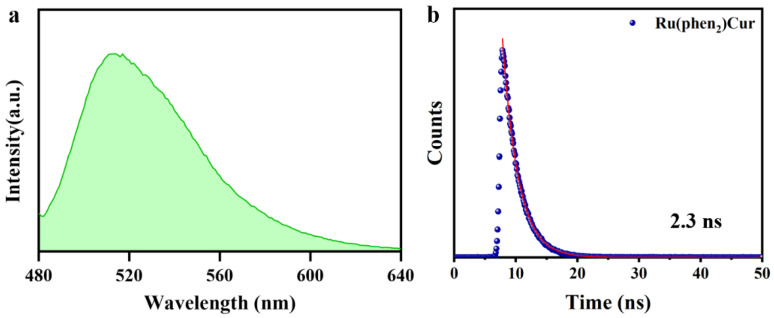
Characterizations of Ru(II)-coumarin complexes. (**a**) Fluorescence spectra of Ru(II)-coumarin complexes with the λ_ex_ = 450 nm. (**b**) Fluorescence lifetime of Ru(II)-coumarin complexes.

There was also huge disparity in their luminescent properties, showing great potential as selectivity switching probes. The ultraviolet-visible absorption spectra and fluorescence spectra of RuCM NP and free molecules were analyzed. The absorption peaks of the RuCM NP at 286 nm belonged to the π → π* electron transition. The absorption bonds of the RuCM NP from 300 to 500 nm may be the charge transfer between the ligand and the metal mixed with the metal−ligand charge transfer transitions (MLCTs) and ligand−ligand charge transfer transitions (LLCTs). The RuCM NP had the same absorption peaks as free Ru(II)-coumarin complexes, albeit with variable peak heights (Figure 1c and Supplementary Figure S1). The fluorescence properties of Ru(II)-coumarin complexes were investigated by photoluminescence spectrometry as depicted in Figure 1d. Upon irritation, RuCM NP displayed double emission peaks at 520 nm and 660 nm in aqueous solution, which was belonged to LLCTs and MLCTs, respectively. In contrast, the DMSO solution of complex displayed a single fluorescence emission peak at 520 nm and short fluorescence lifetime (2.3 ns) belong to emission from LLCTs (Figure 2a,b) [21,30,31]. The fluorescence emission peaks at 660 nm disappeared due to the fact that the disassembled nanoparticles reduced MLCTs, thereby weakening the fluorescence emission, and the fluorescence emission peak at 520 nm in DMSO solution was significantly enhanced compared with that of the RuCM NP in water. Specially, the fluorescent property could be switchable from red to green through the dynamic disassembly of nanoprobes. The green fluorescence could be enhanced during the process of nanoprobe disassembly. Ru(II)-coumarin complexes also showed a higher QY (9.84%) than nanoparticles(0.34%) by introducing a coumarin ligand. The differences in the fluorescence emission properties helped distinguish the signals of two states of the probe in cells.

**Figure 3:**
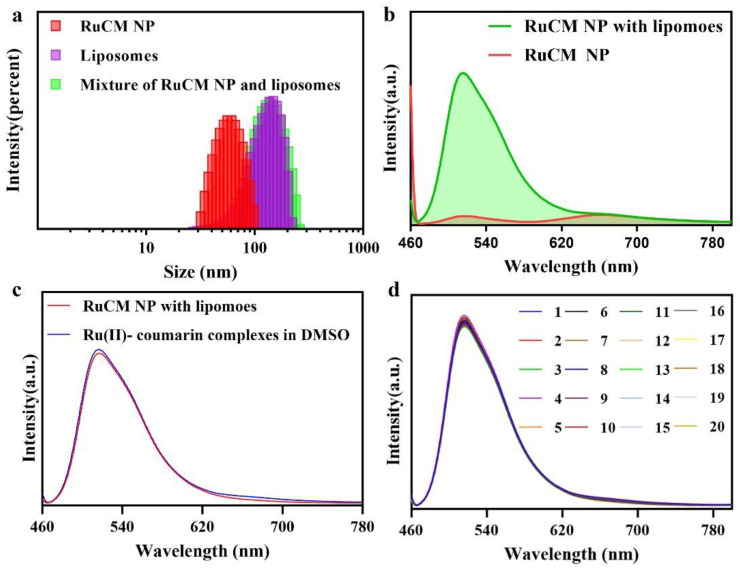
Liposomes selective labelling of RuCM NP. (**a**) DLS of the RuCM NP, liposomes and the mixture of liposomes and RuCM NP. (**b**) Fluorescence spectra of RuCM NP and the mixture of liposomes and RuCM NP. (**c**) Fluorescence spectra of RuCM NP and the mixture of liposomes and Ru(II)-coumarin complexes in DMSO; (**d**) photostability studies of Ru(II)-coumarin complexes after continuous 20 scans.

Selective labelling of cell membrane can reduce the interference of background fluorescence. When the dynamic assembly and disassembly of RuCM NP was confirmed, the feasibility of turn-on fluorescence imaging in lipophilic phase was further investigated. The amphiphilic phospholipid bilayers are the main composition the of cell membranes. Commercial liposomes were used to imitate the cell membrane structure for the study of selective cell membrane imaging. As shown in Figure 3a, DLS results revealed that the RuCM NP could disintegrate rapidly after mixing with liposomes. The red fluorescence of RuCM NP disappeared, and green fluorescence of complexes was robustly enhanced as shown in Figure 3b. In contrast, the fluorescence signal of probe did not change significantly after mixed with PLGA nanoparticles (Figure 3c). These results together demonstrated that RuCM NP could selectively insert in phospholipid bilayers to mark the cell membranes. Further the results found that RuCM NP-labelled liposomes have strong fluorescence stability, overcoming the photobleaching defect of commercial organic small molecule dyes (Figure 3d).

**Figure 4:**
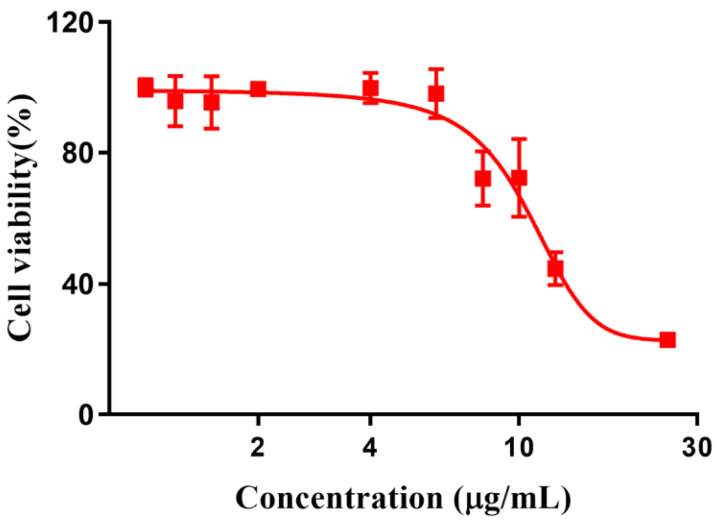
Cytotoxicity of Ru(II)-coumarin complexes.

Ascribed to the switchable and tunable fluorescence emission, RuCM NP was further performed to investigate the selective imaging of cell membranes. Before being applied to this study, the cytotoxicity of RuCM NP was evaluated by CCK-8 assays (Figure 4).

**Figure 5:**
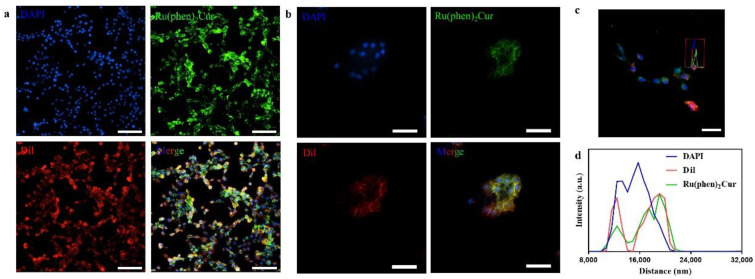
Luminescence confocal microscopy images of 4T1 cell membrane. (**a**) Fluorescence imaging of 4T1 cells stained with DAPI (blue), Ru(II)-coumarin complexes (green) and Dil (red). Scale bar: 100 μm. (**b**) Fluorescence imaging of 4T1 cells stained with DAPI (blue), Ru(II)-coumarin complexes (green) and Dil (red). Scale bar: 50 μm. (**c**) Fluorescence imaging of 4T1 cells stained with DAPI (blue), Ru(II)-coumarin complexes (green) and Dil (red). Scale bar: 100 μm; (**d**) The line scanning profiles of the fluorescence intensity in the corresponding confocal images in (**c**).

**Figure 6:**
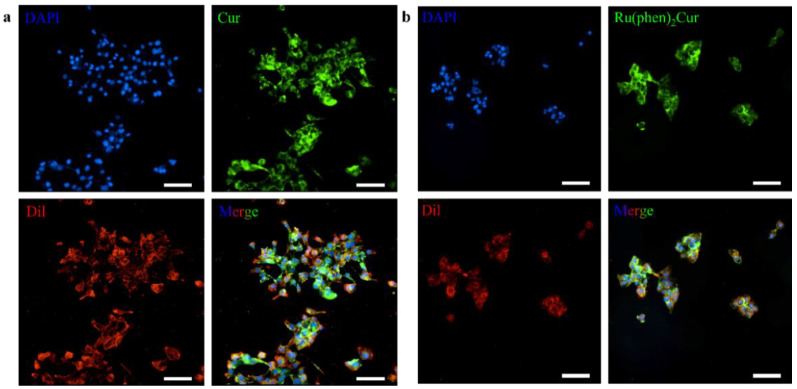
Luminescence confocal microscopy images of 4T1 cell membrane. (**a**) Fluorescence imaging of 4T1 cells stained with DAPI (blue), coumarin 6 (green) and Dil (red). Scale bar: 100 μm. (**b**) Fluorescence imaging of 4T1 cells stained with DAPI (blue), Ru(II)-coumarin complexes molecules (green) and Dil (red). Scale bar: 100 μm.

**Figure 7:**
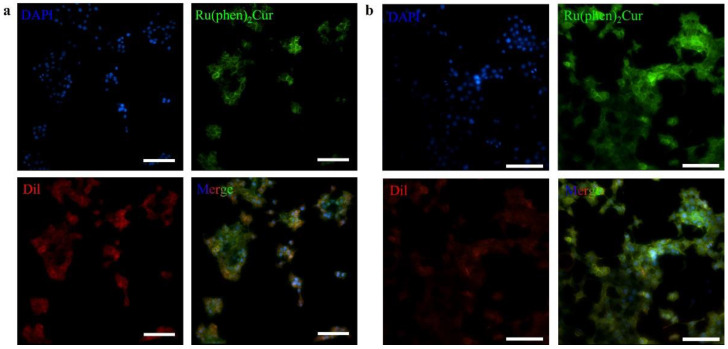
Photostability studies of Ru(II)-coumarin complexes. (**a**) Fluorescence imaging of 4T1 cells stained with DAPI (blue), Ru(II)-coumarin complexes (green) and Dil (red) before laser scans. Scale bar: 100 μm. (**b**) Fluorescence imaging of 4T1 cells stained with DAPI (blue), Ru(II)-coumarin complexes (green) and Dil (red) after laser scans. Scale bar: 100 μm.

The results showed that viability of 4T1 cells were more than 85% after 24 h of incubation with 8 μg/mL RuCM NP and half maximal inhibitory concentration of cell viability was about 10 μg/mL., indicating the good biocompatibility of RuCM NP. Subsequently, 4T1 cells were chosen to perform the cell imaging study. After incubating 4T1 cells with 2.5 μg/mL RuCM NP for 30 min, we observed green fluorescence signal on plasma membrane, indicating that RuCM NP can rapidly disassemble to form free Ru(II)-coumarin complex by the interaction with phospholipid bilayers (Figure 5a). As shown in Figure 5b–d, the green fluorescence signal of the complex was co-localized with the red fluorescence signal of Dil, a commercial cell membrane probe. The Pearson correlation coefficient (R) was 0.743, indicating good selectivity of Ru(II)-coumarin complex to cell membrane. Fluorescence imaging in Figure 6a and the Pearson correlation coefficient (0.461) indicated that coumarin 6 did not display selective cell membrane imaging. Furthermore, cell membrane imaging of the Ru(II)-coumarin complex had worse selectivity than that of the self-assembled Ru(II)-coumarin nanoparticles as shown in Figure 6b, which confirmed the superiority of self-assembled Ru(II)-Coumarin nanoparticles in cell membrane imaging. Generally, many commercial plasma membrane probes failed to achieve long-term imaging due to photobleaching. Subsequently, the photostability of Ru(II)-coumarin complex and Dil was evaluated with continuous excitation and sequential scanned with a confocal microscope. We observed that the fluorescence signal of Dil reduced to only 30% fluorescence intensity within 100 scans. In contrast, the Ru(II)-coumarin complex retained 90% of its original fluorescence intensity, indicating a better photostability (Figure 7). Collectively, these results demonstrated that RuCM NP could disassemble and rapidly inserted into cell membrane due to the specific interaction with phospholipid bilayers. Specially, a nanoprobe with switchable and tuneable fluorescence in the dynamic disassembly process could selectively label cell membranes, reducing background interference.

## 4. Conclusions

In summary, we have reported a self-assembled ruthenium (II)-based nanoprobe, which was able to target the cell membrane for cellular imaging. This nanoprobe interacted with cell membrane and disassembled to molecular dye to achieve the switchable fluorescent for efficient cell membrane imaging. Compared with traditional cell membrane dye, our nanoprobe exhibited stronger stability and lower background interference. Such a switchable nanoprobe also possessed long-term cell membrane imaging. We thus anticipate that this nanoprobe will be powerful for investigating lipid trafficking with optical imaging. The rational design of modular structure of nanoprobe may allow the extension of this strategy to target membranes from specific cells including cancer cell, bacterium, or yeast. Such future efforts should contribute to unraveling the relationship between structure and membrane targeting of nanoprobe, which remain largely unknown.

## Data Availability

The raw data supporting the conclusions of this manuscript will be made available by the authors, without undue reservation, to any qualified researcher.

## Supplementary Materials

The following supporting information can be downloaded at: www.mdpi.com/article/10.3390/pharmaceutics14112284/s1, Figure S1: ^1^NMR of Ru(II)-coumarin complexes; Figure S2: Tyndall effect of Ru(II)-coumarin complexes in DMSO (a) and RuCM NP (H_2_O) (b).
