# Supplementary material for: Self-Assembled Ru(II)-Coumarin Complexes for Selective Cell Membrane Imaging
Source: Pharmaceutics. 2022 Oct 25;14(11):2284. doi: 10.3390/pharmaceutics14112284 (PMC9695855; doi:10.3390/pharmaceutics14112284)
Supplement: Supplementary file 1 [file pharmaceutics-14-02284-s001.zip › pharmaceutics-1963228-supplementary.pdf]

Supplementary Materials: Self-Assembled Ru(II)-Coumarin Complexes for Selective Cell Membrane Imaging

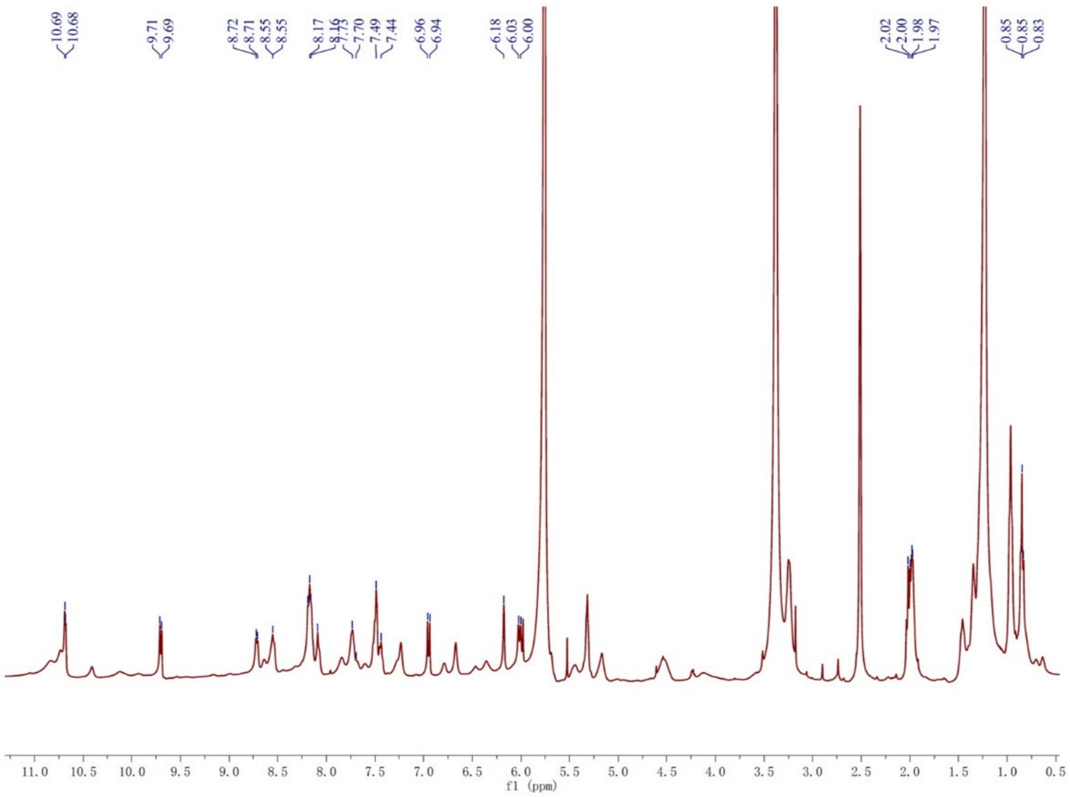

Figure S1. ^{1}NMR of Ru(II)-coumarin complexes.

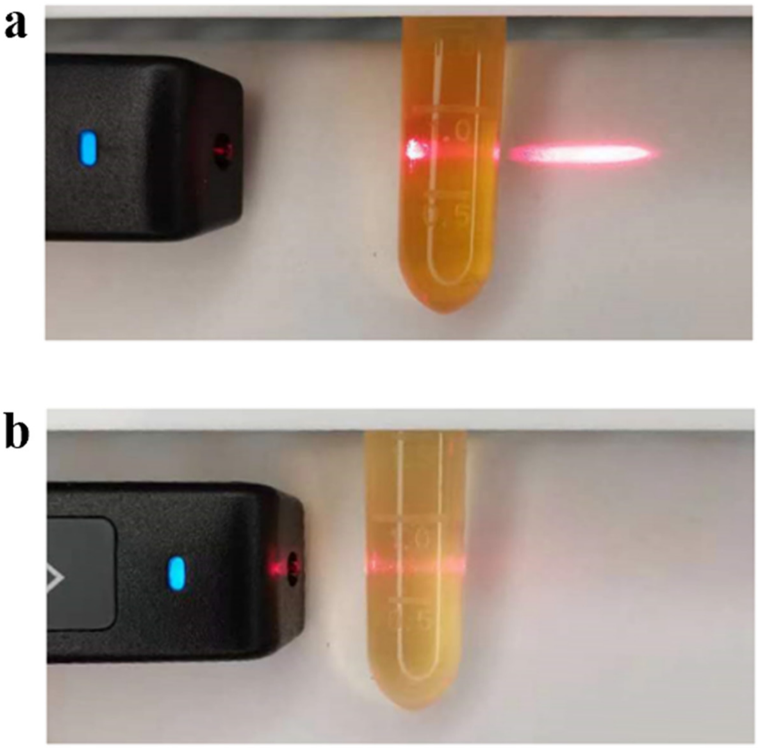

Figure S2. Tyndall effect of Ru(II)-Coumarin Complexes in DMSO (a) and RuCM NP (H_2O) (b).

Tyndall effect exist in polar solvents (H_2O) but vanished in non-polar solvents, which suggesting the hydrophobicity of the free molecule and the form of RuCM NPs in H_2O.
